# Opioid Receptors and Protonation-Coupled Binding of Opioid Drugs

**DOI:** 10.3390/ijms222413353

**Published:** 2021-12-12

**Authors:** Samo Lešnik, Éva Bertalan, Urban Bren, Ana-Nicoleta Bondar

**Affiliations:** 1 Faculty of Chemistry and Chemical Engineering, University of Maribor, SI-2000 Maribor, Slovenia; urban.bren@um.si; 2 Theoretical Molecular Biophysics Group, Department of Physics, Freie Universität Berlin, 14195 Berlin, Germany; eva0bertalan@gmail.com; 3 Faculty of Mathematics, Natural Sciences and Information Technologies, University of Primorska, SI-6000 Koper, Slovenia; 4 Faculty of Physics, University of Bucharest, Atomiștilor 405, 077125 Măgurele, Romania; 5 Forschungszentrum Jülich, Institute of Neuroscience and Medicine and Institute of Advanced Simulation, Computational Biomedicine (IAS-5/INM-9), Wilhelm-Johnen Straße, 52425 Jülich, Germany

**Keywords:** GPCRs, opioid receptors, opioid drugs, molecular dynamics, force field parameters, protonation-coupled dynamics, safe painkillers

## Abstract

Opioid receptors are G-protein-coupled receptors (GPCRs) part of cell signaling paths of direct interest to treat pain. Pain may associate with inflamed tissue characterized by acidic pH. The potentially low pH at tissue targeted by opioid drugs in pain management could impact drug binding to the opioid receptor, because opioid drugs typically have a protonated amino group that contributes to receptor binding, and the functioning of GPCRs may involve protonation change. In this review, we discuss the relationship between structure, function, and dynamics of opioid receptors from the perspective of the usefulness of computational studies to evaluate protonation-coupled opioid-receptor interactions.

## 1. Introduction

Opioid receptors are class A rhodopsin-like G-protein-coupled receptors (GPCRs) expressed in the brain, spinal cord, peripheral neurons, and digestive tract, where they bind peptidic endogenous opioids [1] to mediate cell signaling paths. There are three major subtypes of opioid receptors (ORs): the μ (MOR), δ (DOR), and κ (KOR) receptors; activation of the MOR is implicated in cell signaling for the feeling of pain, and for unwanted side effects related to opioid drug usage [2]. Indeed, targeting the MOR with morphine, fentanyl, or oxycodone, is the most frequently used strategy for treating moderate and severe pain, such as pain associated with cancer or traumatic injury. However, activation of the MOR hyperpolarizes neurons of the central nervous system, which can result in diminished respiratory drive and even death. MOR activation can also inhibit peristalsis and fluid secretion in the digestive system, leading to constipation. Whereas the analgesic effects of opioids often diminish upon continuous use, leading to tolerance, the side effects they cause remain, such that controlling pain requires higher and higher opioid doses -which, combined with the high addiction potential of opioids, can become life-threatening. Only in 2017, opioid overdoses were linked to over 70,000 deaths in the USA [3], and 81% of fatal drug overdoses in Europe [4]. This makes the design of safer analgesics a major research topic.

There are currently several major strategies for the development of safer treatment of pain. The first strategy exploits functional selectivity of opioid receptors, such that downstream signaling via G-proteins, instead of β-arrestins, would be activated. A difficulty with this strategy is that respiratory driver neurons in the brainstem appear to respond to opioids mainly via G-protein signaling [5,6]. Moreover, ligands developed as selective G-protein activators still cause side effects and have a high potential for dependence and abuse. Oliceridine, initially considered a promising partial agonist for selective binding, and which has a better safety profile than the full agonist fentanyl [7,8], lacks selective binding [9]; the observation that ligands with low intrinsic efficacy have an improved side effect profile support the hypothesis that intrinsic activity largely governs unwanted side effects [9].

Other strategies include the enhancement of endogenous opioid mechanisms by inhibiting degradation of endogenous peptides, or using positive allosteric modulators to increase the affinity and efficacy of endogenous opioids when they bind to the orthosteric opioid receptor binding sites [10,11]—but this latter strategy has the disadvantage that allosteric ligands can be species- and receptor-selective, such that a compound active at, e.g., mice receptors, lacks activity towards the human receptor [12].

A very promising strategy in developing safer opioids is to restrict the activity of opioids to peripheral sensory neurons in injured and inflamed tissue [13]. Since peripheral MORs are largely responsible for the overall analgesic effects of opioids, selectively targeting opioids to these MORs could enable safer treatment of pain [1,14,15,16,17,18], although analgesic effects might be insufficient in the case of very painful pathologies, and opioid drugs will need to be protonated in order to bind to the MORs.

Modern in silico methods can provide a detailed picture of opioid binding to GPCRs, and thus help guide the development of safer opioid drugs. We review here opioid receptor function with a focus on recent developments from in silico studies of the MOR and opioid drugs that bind to the MOR.

## 2. Opioid Receptor Function

**Figure 1:**
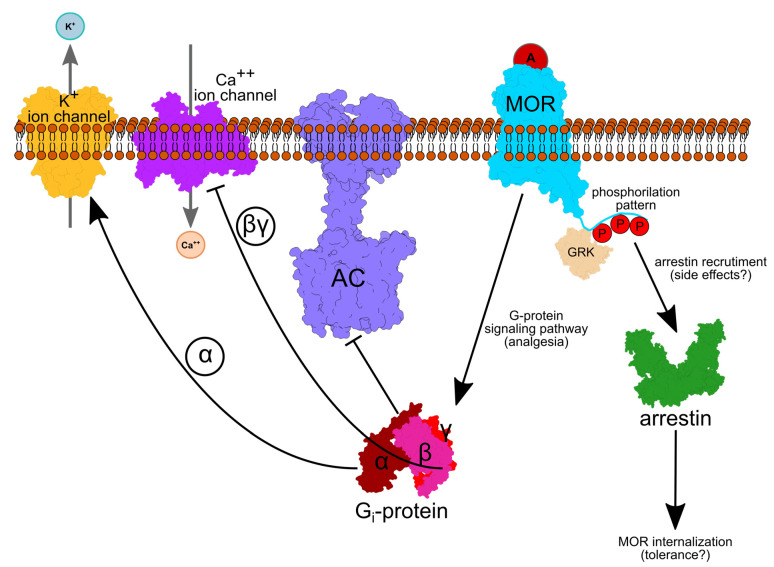
The cellular path of opioid analgesia. An agonist (red circle A), such as morphine, activates the MOR. Depending on the phosphorylation (P) pattern, agonist binding leads to G_i_-protein and/or arrestin-based signaling. GRK proteins add phosphate groups to specific Ser/Thr amino acid residues. An active, GTP-bound G_α_ inhibits Adenylate Cyclase (AC), whereas G_βγ_ inhibits inward Ca^++^ and increases outward K^+^ current, such that the neuronal membrane is hyperpolarized. Arrestin activation leads to internalization of the receptor, followed by degradation or recycling. Protein depictions were created with UCSF Chimera 1.14 [23], whereas the lipid membrane, text and arrows were added using Inkscape 1.0.

GPCRs are seven-helical trans-membrane proteins that mediate communication between cells and their environment [19]. In response to an extracellular signal, GPCRs change conformation to an activated form that binds the cytoplasmic, heterotrimeric G protein (Figure 1). Exchange of GDP with GTP in the G_α_ subunit associates with dissociation of the G_α_ subunit from G_βγ_. G_α_ then inhibits adenylyl cyclase—thus, it inhibits the production of cAMP. G_βγ_ interacts with numerous membrane ion channels, including inwardly rectifying potassium ion channels whose opening lowers neuronal excitability, reducing the propagation of action potentials [20,21]. Ion channels of the Transient Receptor Potential cation channel subfamily V member (TRPV1), which is implicated in various inflammatory hyperalgesia conditions, is likewise inhibited by opioids in dorsal root ganglion neurons [22].

Various G-protein-coupled Receptor Kinases (GRKs) can phosphorylate the C-terminal tail of MOR; depending on the phosphorylation pattern, arrestin might be recruited, which could prevent G-protein coupling to the OR and cause receptor internalization via endocytosis [24], which could lead to opioid drug tolerance [25].

## 3. Architecture of the MOR, and Opioid Binding to the MOR

**Figure 2:**
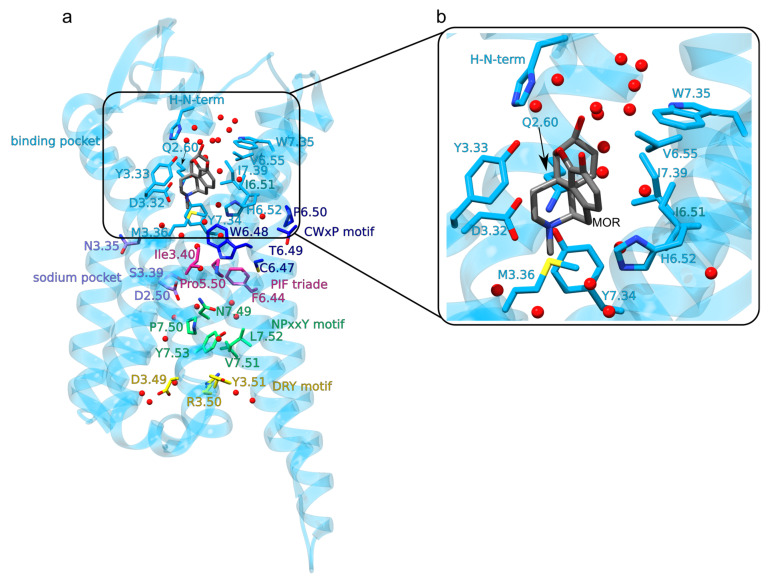
Architecture of the MOR. The seven TM helices inter-connect via the three extracellular loops ECLs 1-3 and the three intracellular loops ICLs 1-3. The disulfide bridge between C3.35 and C45.40 connects TM3 to ECL2. (**a**) Mouse MOR bound to morphine (PDB ID: 5C1M) [32]. The opioid binding site and amino acid residues part of the conserved GPCR motifs are shown as colored bonds. The image was prepared using UCSF Chimera 1.14 [23] and Inkscape 1.0, and is based on the structure of the active MOR (PDB ID: 5C1M) [32]. (**b**) Close view of interactions at the opioid-binding site. We removed from the structure the native crystal ligand BU72. We placed the morphine molecule into the binding site by overlapping it onto the BU72 [32]; for the structural overlap we used the common atoms of the pentacyclic moiety of BU72 and morphine. Water oxygen atoms within 3.5 Å of morphine or protein groups are shown explicitly as red spheres.

**Figure 3:**
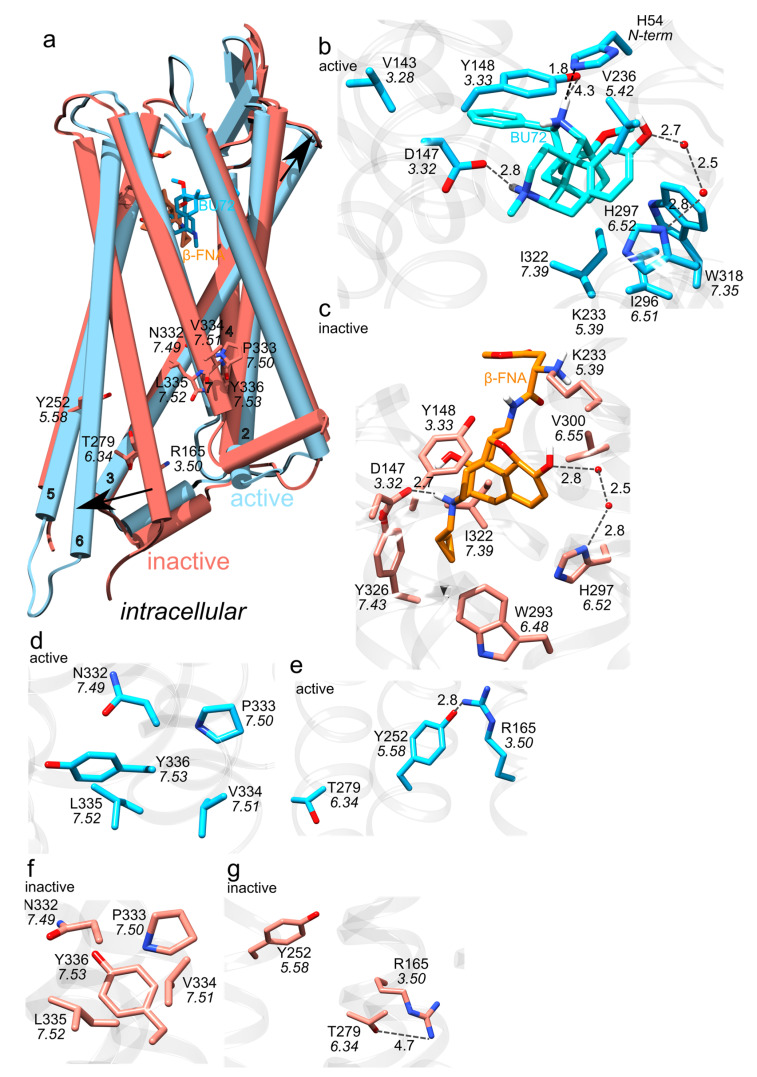
Active vs. inactive MOR. (**a**) Overlap of the structures of active-MOR (blue) and inactive-MOR (red); three-dimensional structures are from PDB ID:5C1M [32] and PDB ID:4DKL [33], respectively. The black arrows indicate that TM3 and TM6 have distinct orientations in the two protein structures. (**b**,**c**) Close view of binding site interactions in structures bound to the agonist BU72, vs. the antagonist β-FNA. Blue carbon bonds represent interactions in the active receptor, and orange, in the inactive receptor. Grey dotted lines represent H-bond interactions. (**d**,**f**) Close view of the NPxxY motif in active (blue bonds) vs. inactive MOR (orange bonds). (**e**,**g**) Close view of the ionic lock. R3.50 interacts with T6.34 in inactive MOR (orange bonds), vs. with T5.58 in active MOR (blue bonds). Molecular graphics were prepared using UCSF Chimera 1.14 [23] and the text was added with Inkscape 1.0.

Class A GPCRs, to which the MOR belongs, share conserved motifs, called molecular switches (Figure 2a)—these are amino acid residues whose interactions change upon activation of the GPCR [26]. After activation upon agonist binding to the extracellular orthosteric side of the receptor (Figure 2b and Figure 3b) the cytoplasmic DRY motif (D3.49, R3.50 and Y3.51 in the Ballesteros and Weinstein numbering scheme [27]) binds to the G-protein (Figure 1, Figure 2a and Figure 3e,g) [28,29]. This involves structural rearrangement at the NPxxY motif (N7.49, P7.50, and Y7.53): whereas in the inactive MOR (Figure 2a and Figure 3c) the DRY and NPxxY motifs are disconnected from each other (Figure 3f,g), in active MOR NPxxY-Y7.53 reorients and water bridges to Y5.58, which interacts with DRY-R3.50 (Figure 3d,e) [26,28,30]. W6.48 of the CWxP motif (C6.47, W6.48, and P6.50, Figure 2a) relocates and approaches P5.50 of the PIF motif (P5.50, I3.40, and F6.44) [28]. The sodium binding pocket (D2.50, N3.35 and S3.39 in Figure 2a) is thought to help stabilize the inactive receptor [31].

Several three-dimensional structures were solved for the mouse MOR (Figure 2 and Figure 3): MOR bound to the agonist BU72 [32], inactive MOR bound to the morphinan antagonist β-funaltrexamine (β-FNA) [33] (Figure 3a), and active MOR bound to G_i_ or nanobody [34]. Compared to the inactive MOR, in active MOR the intracellular part of TM6 has moved away from the central receptor pore, and TM3 reoriented closer to the extracellular side (Figure 3a).

### The Opioid Binding Site of the MOR

The ligand-binding site of the MOR (Figure 2b), located at the extracellular site, is lined by about the same number of hydrophobic and hydrophilic protein groups, and contains several waters (Figure 2b and Figure 3b,c) [35]; this could help explain why the MOR can bind ligands with different size and flexibility, such as large peptidic endogenous opioids, the rigid morphine molecule, or the flexible fentanyl. Except for the diterpene herkinorin [36], ligands that bind to the MOR have a charged amine group that salt-bridges to D3.32—and this salt bridge is thought essential for the activation of the MOR (Figure 2b and Figure 3b) [37,38].

**Figure 4:**
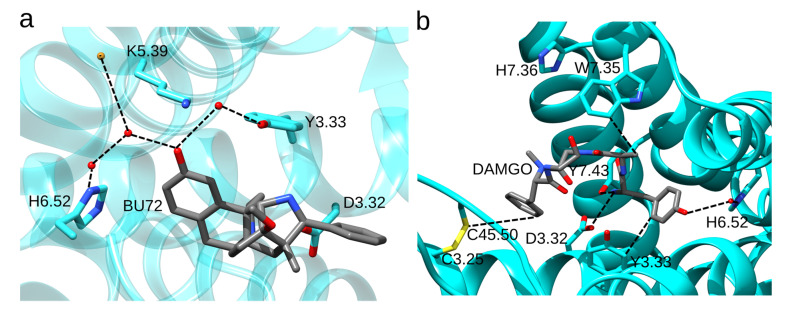
Ligand-protein interactions in static structures of the MOR. (**a**) Water-mediated H-bond network of H6.52 in the structure of BU72-bound MOR. The agonist BU72 H-bonds to H6.52 via two waters. The H-bond network includes K5.39 and Y3.33. D3.32 is close to the ligand. The molecular graphics is based on structure PDB ID:5C1M [32]. (**b**) Binding site interactions of DAMGO. Protein groups shown have at least one side chain atom within 4 Å of DAMGO. The closest atoms between DAMGO and MOR groups shown are connected with dotted lines. Molecular graphics based on PDB ID:6DDF [34] were prepared using UCSF Chimera 1.14 [23] and the text and dotted lines were added with Inkscape 1.0.

Three-dimensional structures [32,33,34] and docking studies performed on the static structure of the MOR with the GOLD software and GoldScore [39] as the scoring function suggested that H6.52 has a water-mediated bridge with the phenolic group of *N*-methylmorphinans and *N*-phenethyl opioid ligands (Figure 3b,c) [40]. In the structure of the active-like, BU72-bound MOR (Figure 4a), H6.52 is part of a water-mediated H-bond network with K5.39 and Y3.33 [32]. Interactions between H6.52 and opioid drugs may depend on both the drug (mutating the His sidechain to Ala reduces the binding affinity for the agonist [D-Ala2, *N*-MePhe4, Gly-ol]-enkephalin (DAMGO) and antagonist naloxone, but leaves the binding affinity for fentanyl largely unchanged [41]) and on the protonation state of H6.52 (fentanyl binds deeper into the binding site when H6.52 has δ-protonation [42]). H-bonding between D3.32 and Y7.43 depends on the ligand, being more frequently sampled in the presence of fentanyl than morphine [43,44].

In the crystal structure of the active-like MOR bound to the agonist DAMGO, DAMGO interacts with W7.35 (Figure 4b) [34]. The W7.35A mutant has reduced binding affinity for DAMGO and morphine, but almost wild-type binding affinities for fentanyl and fentanyl derivatives [45,46]. MD simulations indicated direct interactions between W7.35 and morphine, morphine-like ligands BU72 and β-FNT, DAMGO, and oliceridine [32,44,47,48], but not with fentanyl. The W7.35L mutation reduces morphine and fentanyl binding 5-fold and, respectively, 2-fold [49]. Adjacent to W7.35, H7.36 lacks direct interactions with the ligand (Figure 4b), however, its mutation to Ala reduces somewhat agonist binding [42]. Y3.33 has direct contact with DAMGO (Figure 3b); when Y3.33 was mutated to Phe, the MOR had a 2-3-fold smaller binding affinity for morphine and fentanyl [34,50,51]. Y7.43F cannot bind DAMGO and has diminished binding of morphine and fentanyl [52]. Mutating the conserved C3.25 and C45.50 to serine abolishes opioid binding, which indicates that the disulfide bond enables high-affinity opioid binding (Figure 4b) [53].

Interpreting the observations above on the binding of various opioid drugs to wild-type vs. mutant MOR is difficult due to the scarce information on how various opioid drugs bind to the MOR at room temperature in a fluid lipid membrane environment. For example, even in the case of morphine, most of which consists of a rigid ring structure, quantum mechanical calculations suggest that several conformations could be sampled at room temperature [54]. Moreover, whereas a binding pose for morphine can be derived in silico using the structure of MOR bound to the morphine-like BU72 [47], binding poses for the highly flexible fentanyl and fentanyl-like molecules are challenging to derive with docking, and there is a lack of consensus about details of fentanyl-MOR interactions [42,43,44,55,56,57].

## 4. Opioid Drugs and How They Bind to the MOR

**Figure 5:**
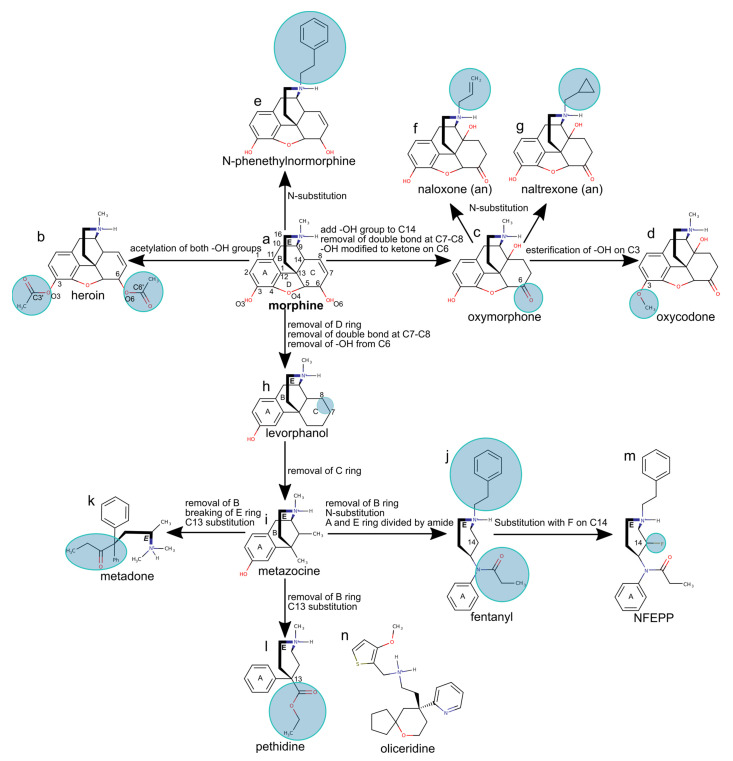
Design of selected morphine-based opioid compounds. We use the morphine molecule (**a**) to introduce the overall numbering and ring labels. In parantheses, “an” indicates an MOR antagonist; all other opioid drug molecules are agonists. Circles highlight the addition or substitution of moieties. The structure of (**b**) heroin, (**c**) oxymorphone, (**d**) oxycodone, (**e**) N-phenethylnormorphine, (**f**) the antagonist naloxone, (**g**) the antagonist naltrexone, (**h**) levorphanol, (**i**) metazocine, (**j**) fentanyl, (**k**) metadone, (**l**) pethidine, (**m**) NFEPP. (**n**) Oliceridine is an agonist unrelated to morphine. Chemical structures were drawn using MarvinSketch 19.4, developed by ChemAxon and assembled using Inkscape 1.0.

Morphine (Figure 5a), a natural opioid obtained from the poppy *Papaver somniferum*, was first isolated as a pure compound in the early 1800s [58,59]. As morphine is poorly absorbed orally, it had limited use before the invention of the hypodermic syringe in 1853. Direct injection into the blood supply showed that morphine has potent analgesic and sedative effects; however, it has severe side effects [60], including respiratory depression, and a high potential for addiction.

Acetylation of the hydroxyl groups of morphine gives heroin (Figure 5b), an opioid drug more active than morphine, but with high addiction potential due to the fast onset of euphoria after intravenous injection. Once it passes the blood-brain barrier, heroin is metabolized to morphine, which then binds to MOR in the brain [59].

Structure-activity relationship studies suggested that essential for the analgesic activity of morphine derivatives are the phenol OH group on C3, the aromatic ring, and the tertiary amine, which is ionized as the drug interacts with the MOR [61]. Adding a hydroxyl group to morphine at the contact of ring B and C on atom C14, as in oxycodone and oxymorphone (Figure 5c,d), increases activity. Docking studies performed with the GOLD software [39] and molecular dynamics studies suggest that the C14 hydroxyl group can H-bond with D3.32 and Y7.43, thereby additionally stabilizing the ligand [40].

**Figure 6:**
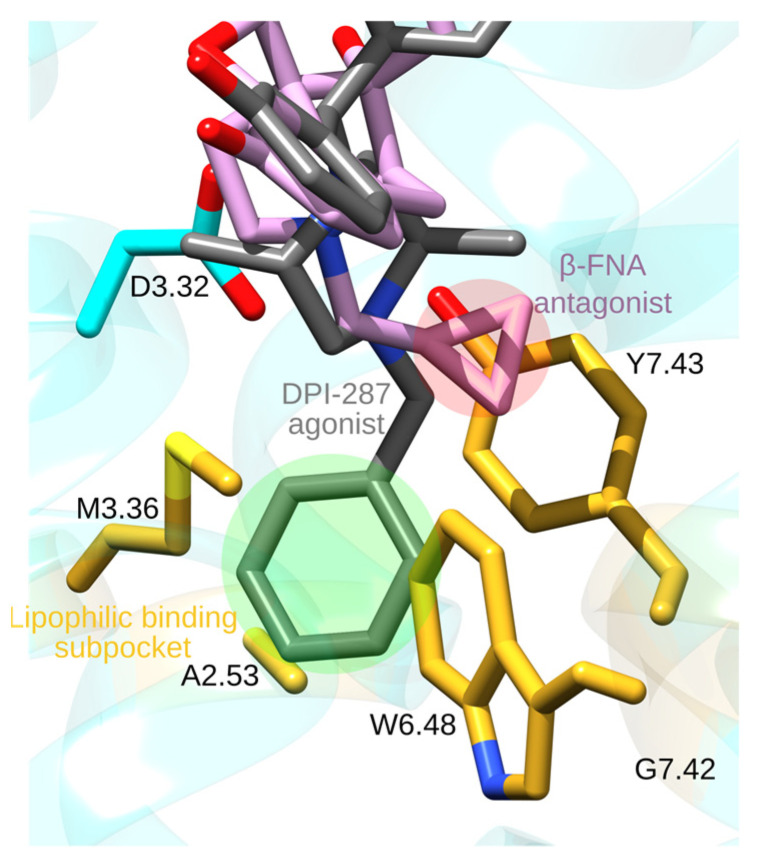
Binding pocket of opioid receptors. The molecular graphics, generated with UCSF Chimera 1.14 [23] and Inkscape 1.0 to add text and highlights, is based on the crystal structure of the DOR bound to the agonist DPI-287 (PDB ID:6PT3) [62]. We generated a structure of the DOR bound to the agonist β-FNA by using UCSF Chimera MatchMaker tool [23,63] to superimpose the DOR to that of the β-FNA-bound MOR (PDB ID: 4DKL). This suggests that the agonist molecule (green highlight) binds deeper than the antagonist (red highlight).

*N*-phenetylnormorphine, obtained from morphine by adding a phenethyl group to the amine N1 atom (Figure 5e), has a 14-fold higher binding affinity than morphine, which could be due to additional contacts made by the *N*-phenethyl group with MOR groups of the lipophilic subpocket. This suggestion is supported by the structure of the active-like DOR bound to the agonist DPI-287; DPI-287 has an *N*-benzyl moiety whose *N*-benzyl group binds at a pocket delineated by A2.53, M3.36, W6.48, G7.42, and Y7.43 (Figure 6) [62].

Adding a smaller allyl or cyclopropylmethyl group to the amine group of oxymorphone leads to antagonists, such as naloxone or naltrexone (Figure 5f,g). The structure of MOR bound to the morphinan antagonist β-FNA [33], which has a cyclopropylmethyl group similar to naltrexone, shows that the cyclopropylmethyl group binds at the same protein site as the *N*-benzyl group of DPI-287, though details of the interactions can be slightly different (Figure 6). Short *N*-methyl substituents are thought to allow the opioid drug to interact with D3.32, which favors activity, whereas larger allyl or cyclopropylmethyl substituents might have steric conflicts with the binding pocket, and thus weaker interactions with D3.32 and antagonism [64]. Longer substitutions, such as phenethyl or benzyl might have favorable van der Waals interactions with the MOR (Figure 6), which stabilizes interactions with D3.32 and provides agonistic activity to the drug [64].

The complete, rigid pentacyclic carbon skeleton of morphine is not necessary for analgesic activity, and morphine can be simplified to obtain synthetic drugs that are easier to produce and have the same activity as morphine or even higher [65,66]. Removing the oxygen bridge (ring D in Figure 5h), the hydroxy group at atom O4, and the C7=C8 double bond, gives morphinans, such as levorphanol—an opioid drug about five times more active than morphine. Removing rings C and D of morphine (Figure 5i) gives benzomorphans, such as metazocine, whose activity is similar to morphine.

A particularly important simplification of morphine consists of removing rings B, C and D to obtain 4-phenylpiperidines, such as pethidine and 4-anilinopiperidines. The latter class of opioids includes fentanyl (Figure 5j), which is 100 times more potent than morphine [67] and has a high affinity to the MOR [68]. However, fentanyl and fentanyl derivatives have high addiction and respiratory depression potential, which restrict their usage. Methadone, obtained from morphine by removing rings B, C, D and breaking ring E into an acyclic amine (Figure 5k), has an activity similar to morphine, but with fewer side effects; for this reason, methadone is often used to treat opioid addiction [69]. Removing ring B of metazocine and substituting the hydrogen with ethyl carboxylate results in pethidine (Figure 5l), an opioid with a potency about one-eighth of that of morphine, but with a more rapid onset and a shorter duration of action, and which is primarily used in childbirth [70].

*N*-(3-fluoro-1-phenethylpiperidin-4-yl)-*N*-phenylpropionamide (NFEPP), which is a derivative of fentanyl fluorinated on the piperidine moiety (Figure 5m) is a novel type of opioid that is of interest because studies on animal models suggest NFEPP is safer compared to classical opioids, possibly due to selective activation only in peripheral tissue [17,18,71].

Oliceridine (Figure 5n) was discovered by screening for MOR activity [72]. Oliceridine functions as a biased agonist initially thought to bind to the MOR and potentially activate the G-protein path selectively (Figure 1), with reduced recruitment of β-arrestin and fewer side effects [73]. The side effects of oliceridine are now under debate, particularly its potential to cause respiratory depression [9].

## 5. pH-Dependent Binding of a Fluorinated Fentanyl Derivative

Opioids produce their full painkilling effect by acting on both the central (CNS) and peripheral nervous systems [20,74], and their side effects are largely caused by action on the former. Opioid action on peripheral neurons is exploited in the management of painful conditions, such as arthritis, neuropathy, surgical wounds, or cancer [75].

A promising approach to reduce side effects associated with opioid action on the CNS is to restrict opioid activity only on the peripheral sensory neurons in, e.g., inflammation and cancerous tissue [13] that can associate with low pH of 5.5–7.0, as compared to 7.4 in healthy tissue [76], and with upregulation of ORs and their signaling pathways [13,21]. Injury and inflammation are associated with the proliferation of nerve terminals that have ORs, and easier access to opioid drugs due to disruptions of the perineural barrier [77]. Low extracellular pH might enhance inhibition of calcium ion channels by endorphin and morphine (Figure 1) [78]. The pKa of opioid drugs is typically above 7.5 [79,80], i.e., opioid drugs are positively charged at both physiological pH, as well as low pH typical for inflammation. The fluorinated fentanyl derivative NFEPP (Figure 5m) has a pKa of 6.82—which is significantly lower than the pKa of 8.43 for fentanyl [16]. In vitro tests suggested that NFEPP selectively activates MOR at low pH environments [71], it is thought that NFEPP preferentially binds to MORs in peripheral acidic tissue [16].

In vivo tests suggest NFEPP gives effective dose-dependent analgesia with fewer side effects than fentanyl for persistent or acute inflammatory pain [16], a good safety profile in neuropathic pain and inflammatory bowel disease models [81], and reduces cancer-associated pain [18]. Contrary to fentanyl, NFEPP appears to lack inhibition of peristalsis as a side effect [82]. Overall, the safety profile of fluorinated fentanyl derivatives, including NFEPP [79], depends on the pKa value of the drug, with lower pKa being associated with fewer side effects.

Molecular mechanisms by which low pH impacts drug binding to ORs are poorly understood. At low pH, protonation states of titratable amino acid residues of the MOR might change, which could influence the internal H-bond network [41]. At pH = 6.0 there is a reduced binding of naloxone and DAMGO, however, fentanyl appears to have the same binding at low and physiological pH [41]. One amino acid residue whose protonation could change at low pH is D2.50 (Figure 2a), as the D2.50N mutant, which might hint about the MOR with protonated D2.50, has reduced binding of DAMGO, morphine, and naloxone [37]. In another class A GPCR, bovine rhodopsin protonation of D2.50 is thought to be involved in receptor activation [83].

## 6. Force-Field Parametrization of Opioid Drugs

Classical mechanical Molecular Dynamics (MD) simulations would allow us to probe how the MOR responds to the binding of various ligands. Importantly, by an appropriate choice of the protonation of the ligand and titratable protein groups, MD simulations may be used to derive clues about protonation-coupled protein dynamics.

MD simulations are performed based on a potential energy function, or force-field that uses several predetermined parameters denoted as force-field parameters. The accuracy of the parameters is essential for the MD simulations to be reliable.

**Figure 7:**
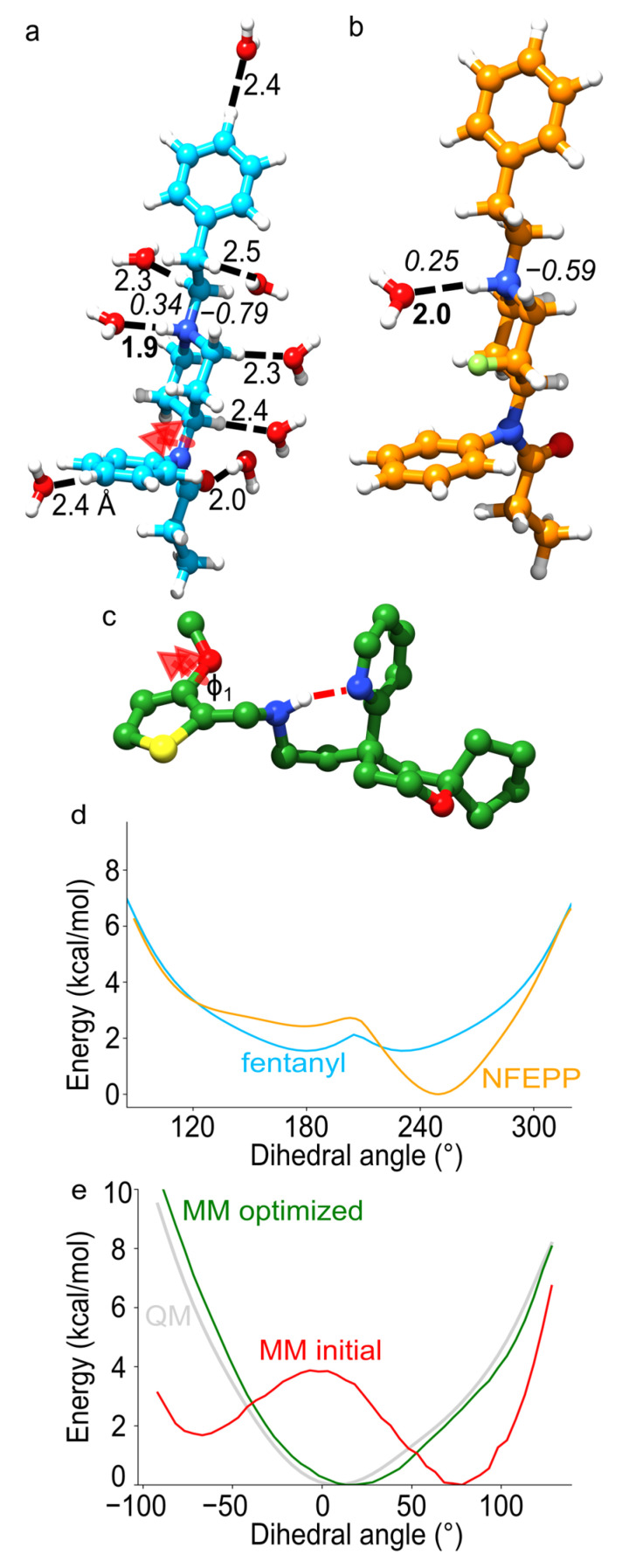
Force-field parameterization of fentanyl, NFEPP and oliceridine. (**a**) Illustration of QM interaction distances (regular fonts) and atomic partial charges (italic fonts) for selected sites of fentanyl. Note that the protonated amine can H-bond to water. The arrow indicates the bond for which we illustrate in panel d the torsional energy profile. (**b**) H-bond distance and partial charge values for the charged amine group in NFEPP. (**c**) Coordinate snapshot from MM MD of oliceridine [88] illustrating an internal H-bond (red dotted line) sampled between the piperidine and secondary amine nitrogen atoms. Dihedral angle Φ_1_ is flexible and required force-field parametrization. (**d**) Illustration of the potential energy scans for a bond twist parametrized for fentanyl and NFEPP. Potential energies, taken relative to the energy of the equilibrium geometry, were computed with MM parameters derived in Ref. [87]. (**e**) Potential energy scans computed for angle Φ_1_. The initial MM profile is colored red, QM target profile, gray, and MM optimized, green. Panel e is based on data published in Ref [88]. Molecular graphics in panel a, b and c were created with UCSF Chimera 1.14 [23], whereas the text, arrows and dotted lines were added with Inkscape 1.0. Plots in d and e were generated using Python’s *matplotlib* package [89].

The CHARMM General Force Field (CGenFF) protocol to parametrize drug-like molecules ensures that the parameters for the drug molecule are compatible with the CHARMM force-field parameters for protein, lipids, and water molecules [84,85,86] (Figure 7). Briefly, the iterative CGenFF procedure uses exhaustive QM calculations that serve as target data for MM—computations include geometry optimization, normal mode analysis, potential energy scans of dihedral angles, and water interaction energies (Figure 7a,b) [84]. This protocol was used to derive force-field parameters for fentanyl and NFEPP [87].

Partial atomic charges are essential for an accurate MM description of non-covalent interactions between drug ligands and the MOR. To obtain reliable values of atomic partial charges for fentanyl and NFEPP, water interaction energies and distances computed with classical mechanics were fitted to the QM counterparts, where QM calculations served as reference (Figure 7a). The MM partial atomic charges were automatically fitted until MM energies and interaction distances agreed with QM values. These computations revealed that, relative to fentanyl, the presence of the fluorine atom in NFEPP alters the partial atomic charges (Figure 7b): Fentanyl has an overall larger positive charge of the amine system, which could allow stronger H-bonding to MOR.

Fentanyl and NFEPP have flexible linkers that connect rather rigid fragments (Figure 5j,m). To correctly describe the torsional profiles of the flexible linkers, potential energy scans were performed with QM, and fitted manually the torsional parameters until MM energy scans agreed with QM (Figure 7d). The potential energy profiles for the torsions of the bond between the (fluoro)piperidine and aminobenzene moieties are distinct in NFEPP vs. fentanyl: The potential energy profile is symmetrical in the case of fentanyl (Figure 7d), as compared to the mode complex, nonsymmetrical profile obtained for NFEPP, suggesting fentanyl and NFEPP likely have different conformational dynamics.

To enable a systematic study of how the MOR responds to the binding of different opioid drugs, a force-field parameters for oliceridine, morphine, and heroin was developed recently [88] (Figure 5a,b,n). Similarly, to fentanyl, oliceridine has flexible linkers that inter-connect largely rigid components. By contrast, morphine and heroin consist of largely rigid fragments. This presented a possible solution to simplify the parametrization procedure and focus on parametrizing torsional potentials for dihedral angles likely to contribute more to structural dynamics at room temperature: first, the dynamics of isolated compounds was probed with QM, the dynamics of selected dihedral angles was inspected, and then the results used to focus the parametrization on soft dihedral angles. Computations performed during parametrization of oliceridine revealed that significant improvement was required for the correct description of torsional potentials involving thiophene bonds—for example, for dihedral angle ϕ_1_ (Figure 7c) the original MM torsional potential had an energy barrier at the same angle value for which QM indicates an energy minimum (Figure 7e). With the corrected torsional potentials and partial atomic charges, a reliable description of the structural properties of oliceridine could be obtained [88].

During MD, the inter-helical region of opioid receptors and other GPCRs is visited transiently by numerous water molecules [90,91]. Thus, upon binding to the MOR, opioid drugs may also interact with internal waters that visit the protein. QM MD simulations of opioid drugs in the presence of water molecules [88] suggested that, during conformational dynamics, water may access the protonated amine group of the drugs. In the case of oliceridine, an internal H-bond observed for the isolated compound (Figure 7c) becomes less likely in the presence of waters [88].

## 7. Classical Mechanical Computations of Opioid Receptors

Rational structure-based drug design for biased agonists remains challenging because ligand-mediated conformational changes that could impact the selection of a specific downstream signaling path remain largely unclear. The selection of specific downstream pathways typically referred to as *biased agonism,* is thought to allow safer side effects profiles [92]. The differentiation of G-protein versus β-arrestin downstream signaling has been extensively researched for the MOR [93,94,95,96,97]. Pairs of *R* and *S* enantiomers of biased MOR agonists are ideally suited for comparison of biased agonism because of differences in bias profiles, i.e., the ability of agonists to active G-protein or β-arrestin downstream signaling are due only to differences in interactions with the receptor. In the case of opioid drugs, *R* enantiomers are biased towards G-protein recruitment, whereas *S* enantiomers activate both G-protein and arrestin pathways [98].

MD simulations [98] indicated that both the *R* and *S* enantiomers of MOR agonists with a piperidine moiety sample a salt bridge with D3.32 and a water-mediated H-bond with H6.52, and hydrophobic interactions with TM2 and TM3. The two enantiomers, however, have distinct interactions elsewhere in the receptor: whereas the *S*-enantiomers interact with Y7.43 and D3.32, R-enantiomers interact with Y3.33 and only weaker with D3.32. Homology and coarse-grained modeling of conformations that could be visited during the activation of the MOR activation were interpreted to suggest that active MOR is stabilized by the binding of the G-protein [99].

The semi-natural compound herkinorin, which is of particular interest as the only known selective MOR agonist without an amino group. Computations on MOR-herkinorin complexes [100] indicated that herkinorin makes water-mediated H-bonding with H6.52, and samples H-bond distances with N2.63 and N3.35 (3.35 Å). The calculated affinity for the binding of herkinorin to the MOR, −11.52 ± 1.14 kcal/mol is close to the experimental value of −10.91 ± 0.2 kcal/mol [101], which suggests the binding pose found with computations is reasonable.

**Figure 8:**
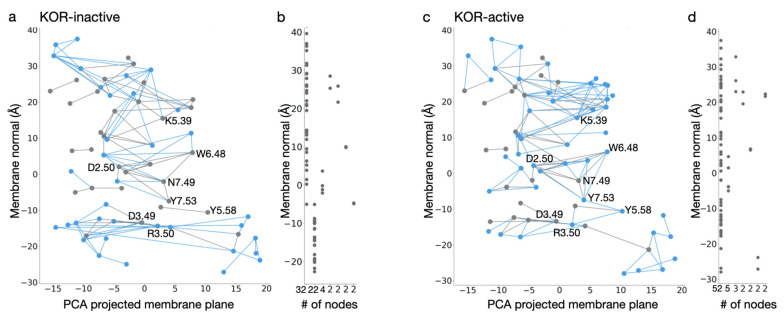
Comparison of water-mediated H-bond network of KOR in inactive and active conformations. (**a**,**c**) Water wire graph of KOR in inactive (**a**) and active (**c**) conformations. Blue lines indicate unique connections in the two structures, whereas gray edges represent H-bonds formed in both conformations of KOR. Nodes that are labeled with BW numbers are part of the conserved GPCR molecular switch motifs (shown in Figure 2a). H-bonds displayed on the graph have a minimum of 40% occupancy in the last 100 ns of the MD simulations. (**b**,**d**) Linear length of continuous H-bond subnetworks along the membrane normal. In the inactive conformation of KOR (**b**), the two longest chains consist of 32 and 22 interconnected protein groups, whereas in active-like KOR (**d**) there is a continuous chain, ~68 Å long, composed of 52 protein groups, that connects the extracellular and intracellular side of the protein. These panels were generated based on data reported in Ref. [90].

Bertalan et al. [90] used MD simulations to probe the motions of ligand-free active vs. inactive KOR, and inactive DOR, and graph-based algorithms to evaluate the dynamics of internal H-bond networks. An internal protein-water H-bond network is interrupted at Y7.53 in the inactive receptor (Figure 8a,b). By contrast, the active-like conformation hosts a continuous network of H-bonds, consisting of 52 amino acid residues, in the transmembrane region (Figure 8c,d). This network, which is sampled ∼40% of the time, extends from K5.39 at the extracellular side to R3.50 at the cytoplasmic side, i.e., from the region where the ligand vs. where the G-protein would bind. Transient H-bonds whose sampling enables a continuous H-bond network throughout the active receptor include Y7.53, Y5.58, and R3.50, where the NPxxY may connect to the DRY motif. The shortest H-bond path between K5.39 and R3.50 passes through all switch motifs characteristic for class A GPCRs and includes amino acid residues from all seven transmembrane helices.

## 8. Future Directions

Due to the increased incidence of painful diseases, especially cancer, and due to the rise in opioid overdoses, novel, safer painkillers for treating moderate and severe pain are urgently needed. However, such attempts have encountered various obstacles, highlighting the need to understand better the molecular mechanisms behind opioid binding and MOR activation [102]. In silico techniques, such as docking, MM and QM simulations, could provide an atomistic description of opioid drug binding to the MOR.

A challenge to MM simulations of opioid drug binding is that opioid drugs are non-standard molecules for which MM force-fields may lack accurate parameters. We presented here recent efforts to derive accurate force-field parameters for five opioid drugs, whose study with MM simulations could provide a glimpse into how the MOR responds to the binding of different drugs. Fentanyl and the fluorinated fentanyl, NFEPP, are drugs distinguished by the presence of a fluorine atom on the latter, yet their safety profiles are rather distinct, and extensive computations performed for force-field parametrization revealed significant differences between the partial atomic charges (Figure 7a,b) and torsional profiles (Figure 7d) for fentanyl vs. NFEPP. The potentially stronger H-bonding at the amino group of fentanyl vs. NFEPP, and the preferential selection of bond twisting in the case of NFEPP (Figure 7d) suggest these two opioid drugs might have distinct interactions with the MOR. In the future, prolonged simulations of the MOR bound to fentanyl vs. NFEPP with different protonation of the drug and of the receptor could provide a glimpse into how protonation change shapes drug-receptor interactions at low pH of interest for pain treatment.

Together with the parameters for fentanyl and NFEPP [87], force-field parameters derived for morphine, heroin, and oliceridine [88] will enable reliable MM simulations to explore how the MOR responds to the binding of distinct opioid drugs. Graph-based methodologies [90,91], as illustrated in Figure 8, would make it possible to identify efficiently internal H-bond networks that contribute to long distance propagation of conformational change upon ligand binding.
